# Motional Narrowing Effects in the Excited State Spin Populations of Mn-Doped Hybrid Perovskites

**DOI:** 10.1021/acs.jpclett.3c03466

**Published:** 2024-03-05

**Authors:** Jonathan Zerhoch, Stanislav Bodnar, James E. Lerpinière, Shangpu Liu, Timo Neumann, Barbara Sergl, Markus W. Heindl, Andrii Shcherbakov, Ahmed Elghandour, Rüdiger Klingeler, Alison B. Walker, Felix Deschler

**Affiliations:** † Physikalisch-Chemisches Institut, Universität Heidelberg, Im Neuenheimer Feld 229, 69120 Heidelberg, Germany; ‡ Walter Schottky Institut, Technische Universität München, Am Coulombwall 4, 85748 Garching, Germany; § Physics Department, TUM School of Natural Sciences, Technische Universität München, Am Coulombwall 4, 85748 Garching, Germany; ∥ Department of Physics, University of Bath, Bath BA2 7AY, U.K.; ⊥ Cavendish Laboratory, University of Cambridge, JJ Thomson Ave, Cambridge CB3 0HE, U.K.; # Kirchhoff Institut für Physik, Universität Heidelberg, Im Neuenheimer Feld 227, 69120 Heidelberg, Germany

## Abstract

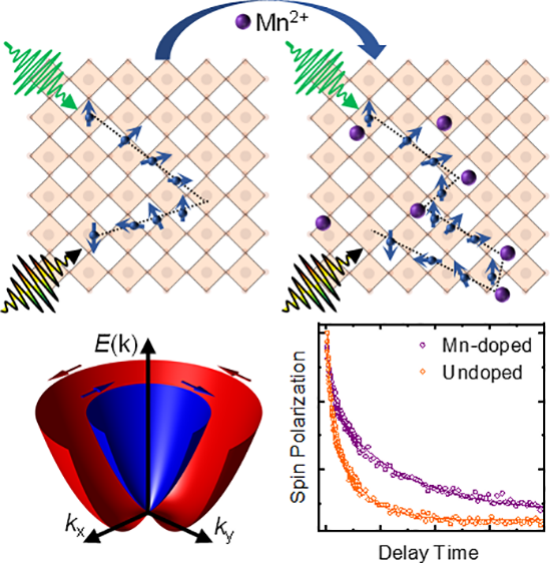

Spin–orbit coupling in the electronic states of solution-processed hybrid metal halide perovskites forms complex spin-textures in the band structures and allows for optical manipulation of the excited state spin-polarizations. Here, we report that motional narrowing acts on the photoexcited spin-polarization in CH_3_NH_3_PbBr_3_ thin films, which are doped at percentage-level with Mn^2+^ ions. Using ultrafast circularly polarized broadband transient absorption spectroscopy at cryogenic temperatures, we investigate the spin population dynamics in these doped hybrid perovskites and find that spin relaxation lifetimes are increased by a factor of 3 compared to those of undoped materials. Using quantitative analysis of the photoexcitation cooling processes, we reveal increased carrier scattering rates in the doped perovskites as the fundamental mechanism driving spin-polarization-maintaining motional narrowing. Our work reports transition-metal doping as a concept to extend spin lifetimes of hybrid perovskites.

Strong spin–orbit coupling (SOC) and long spin lifetimes are desired properties for materials suitable for data storage spintronics and opto-spintronics applications.^1^ Hybrid metal halide perovskites are promising candidates for such applications, since they exhibit strong SOC and are optically active, which enables control of spins via the helicity of light.^2−4^ Further beneficial characteristics of this class of materials are facile and economic solution-based processability, high defect tolerance, outstanding optoelectronic performance, and compositional tunability of optical bandgap and excitonic binding energy.^5−10^ These properties have motivated profound research toward photovoltaic and (spin) light emitting applications.^11−14^ Hence, understanding the spin dynamics and relaxation mechanisms in perovskites is of interest for further optimization of the desired features. For this, extending the current picosecond spin lifetimes of lead halide perovskite thin films toward application-relevant nanosecond time scales is a current objective.

In general, the spin relaxation time in Ruddlesden–Popper layered perovskites is in the single-picosecond range. It increases with the number of layers, driven by the decrease in exciton binding energy with increasing number of layers.^15−17^ On the other hand, in bulk 3D perovskites overall longer spin lifetimes were observed and nanosecond spin lifetime was shown for single crystals.^20,21^ It was identified that spin relaxation mainly happens via the Elliot–Yafet (E-Y)^18^ or the D’yakonov–Perel (D-P) mechanism.^19^

Doping of hybrid perovskite systems with transition metal ions, such as Ni^2+^ or Mn^2+^, has been successfully reported and has generated higher luminescence quantum yields and circularly polarized emission.^22−24^ Dilute Mn-doping of the Ruddlesden–Popper layered perovskite (PEA)_2_PbI_4_ (PEA = C_8_H_12_N^+^) leads to magnetic proximity effects between the excitons and the Mn-spins, which are aligned via a strong external magnetic field, causing circularly polarized emission of up to 13% at 6 T.^23^

It was shown in paramagnetic dilute magnetic semiconductor Ga_(*x*–1)_Mn_*x*_As that the Mn-dopants extend the spin lifetime due to motional narrowing effects, which slow down the precessional spin relaxation via the D-P mechanism.^25,26^

Therefore, we have chosen the 3D perovskite MAPbBr_3_ (MA = CH_3_NH_3_^+^) as the host material to investigate the influence of high-paramagnetic Mn doping on the spin relaxation dynamics.

Here, we employ solution-based manganese doping of polycrystalline thin films of the hybrid perovskite MAPbBr_3_ to realize motional-narrowing interactions between excited charge carriers and Mn-dopants, which we report to increase the spin relaxation time τ_S_ by a factor of 3. Using ultrafast spectroscopy, we gain insights into the detailed spin relaxation mechanisms also at cryogenic temperatures. We demonstrate that the spin relaxation time is extended by motional narrowing effects from increased carrier momentum scattering rates with the phonon modes introduced by Mn^2+^ doping.

To investigate the influence of transition metal doping on excited state spin dynamics and the spin relaxation time in hybrid perovskites, we prepared polycrystalline thin films of pure MAPbBr_3_ and highly Mn-doped MAPbBr_3_ via solution-based processing, where we substituted in the solution the lead precursor Pb(II)-acetate with Mn(II)-acetate in the double-digit percentage range. We refer to this doping concentration as the “nominal” doping level, which differs from the actual Mn^2+^ content that is in the films and which we will specify in the following. Thin films were spin-coated on glass substrates and annealed (full details on sample fabrication are in the Supporting Information). Figure 1a shows the steady state absorption and photoluminescence (PL) spectra of pristine and Mn-doped MAPbBr_3_ with a nominal doping concentration of 50% at room temperature. We find a red shift of the bandgap energy of 30 meV for the Mn-doped compared to the pristine material with a slightly higher Stokes shift in the case of the doped material. X-ray diffraction (XRD) measurements of MAPbBr_3_ with different nominal doping concentrations are shown in Figure 1b and a zoom-in of the (100) and (200) peaks in Figure 1c. Noticeable shifts toward smaller angles (lattice expansion) can be observed for nominal doping concentrations of 50% and 75% with additional small peaks emerging at around 14.12° and 28.40°. The trend of lattice expansion, observed in the XRD measurements, can be interpreted as a sign of interstitial doping of the perovskite crystal with Mn atoms.^27,28^

**Figure 1:**
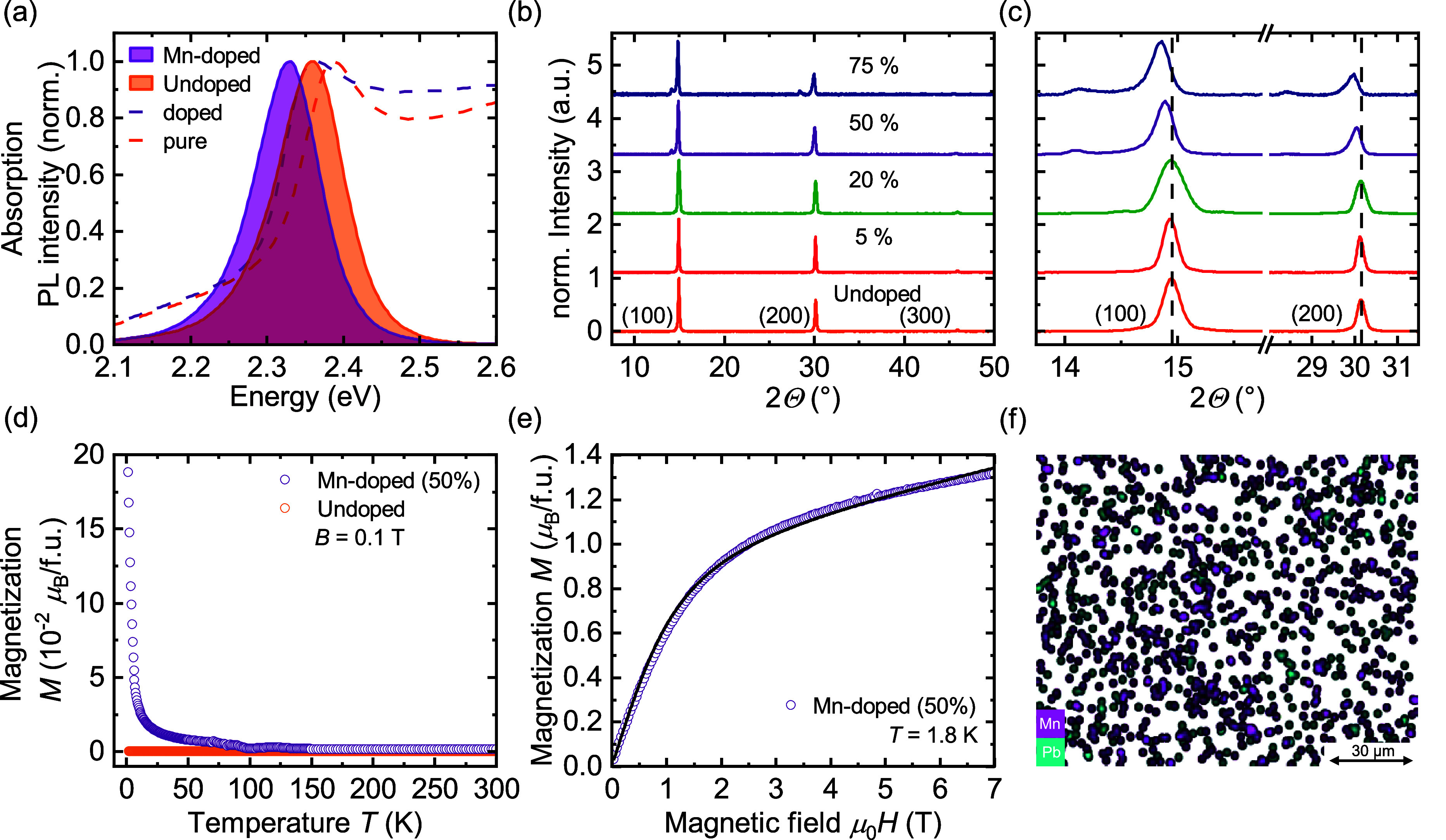
General optical, structural and magnetic characterization of spin-coated thin films of undoped and Mn-doped MAPbBr_3_. (a) Steady-state absorption (dashed lines) and photoluminescence measurements (solid lines with colored areas) of (50%) Mn-doped (purple) and undoped (orange) MAPbBr_3_ at room temperature. (b,c) X-ray diffraction pattern of MAPbBr_3_ for different nominal doping concentrations. (d) Magnetization measurements as a function of temperature in the presence of an external magnetic field of 0.1 T. (e) Magnetization measurements as a function of the external magnetic field at 1.8 K with a Brillouin function fit (black line). (f) Energy-dispersive X-ray (EDX) mapping of lead and manganese for Mn-doped MAPbBr_3_ (50% nominal doping level).

Magnetization (superconducting quantum interference device (SQUID)) measurements show the dominant paramagnetic behavior of our Mn-doped MAPbBr_3_, while undoped MAPbBr_3_ demonstrates only a very weak positive magnetic susceptibility (Figure 1d). Shoulders in the *M*-*T* spectra at around 90 and 125 K suggest the formation of a small amount of antiferromagnetic clusters, such as MnO or MnO_2_. No features of metallic Mn or MnBr_2_ could be found. To obtain the lead–manganese atomic ratio of the doped materials, we analyzed and fitted the magnetization curve as a function of the external magnetic field with a Brillouin function of a *J* = 5/2 system (Figure 1e) (details in the Supporting Information). For a nominal 50% doping concentration, the fit results in a doping concentration of ∼18% per formula unit from Mn^2+^ ions, which contribute to the observed paramagnetic response. Energy-dispersive X-ray spectroscopy (EDX) analysis (Figure 1f) complements these observations from SQUID measurements, indicating the presence of Mn throughout the thin film with fluctuating densities and fluctuating overlap with the Pb atoms. Based on these characterizations, we conclude that under high nominal doping, we obtain a mixed phase material of varying levels of Mn-doped MAPbBr_3_ domains, as well as small amounts of Mn clusters influencing the MAPbBr_3_ crystal structure. The reduced level compared to the nominal amount is likely due to different solubility of the precursors which will especially influence the final concentration during the dynamic spin-coating process. While the precise localization of Mn^2+^ ions within the crystal lattice of hybrid perovskites remains a challenging and unresolved task, our results indicate that a high level of nominal doping concentration leads to electronic and structural interactions between (a subpopulation of) the dopants and the host material. Since our ultrafast investigations of photoexcited charge carrier spin-dynamics probe local photoexcitation dynamics, it is fair to assume that dopant clusters, which are optically inactive, will play a minor role. Thus, we select material systems with a nominal doping concentration of 50% for investigations of spin dynamics, since these offer high doping loading while maintaining good optical properties.

Using ultrafast broadband circularly polarized transient absorption spectroscopy (CTA)^2^ (Figure 2a) at cryogenic temperatures, we investigate the time evolution of spin-polarized charge carriers, injected with an excess energy of 110 meV over the optical bandgap via a circularly polarized pump pulse of 2.41 eV, and probed with circularly polarized white light (1.8–2.4 eV). In lead halide perovskites the valence band consists of hybridized Pb s- and Br p-orbitals with an overall s-like symmetry, and the conduction band of the hybridization of Pb p-orbitals with Br s-orbitals with an overall p-like symmetry.^29^ The conduction band (*l* = 1) further splits up due to the spin–orbit coupling into *m*_*l*_ = ± 1 and *m*_*l*_ = 0 electronic states. The light and heavy electron (*m*_*l*_ = ± 1) degeneracy can be lifted due to a reduction of symmetry (cubic to tetragonal or orthorhombic transition).^29^ Finally, the presence of the Rashba effect lifts the degeneracy of *m*_s_ = ± 1/2 due to the shift of spin-up and spin-down bands along k-directions. For conservation of total angular momentum *J* in optical transitions, the selection rule Δ*m*_*j*_ = ± 1 must be fulfilled for circularly polarized photoexcitation.^30^ Since we study here the spin dynamics close to the bandgap and the splitting between the j = 1/2 and j = 3/2 conduction bands is in the order of 1 eV,^31^ we will focus in the following on the transition between the topmost valence band (VB) and the bottom-most conduction band (CB). J-Polarized excitation of electrons from the VB *m*_s_ = −1/2 (+1/2) state to the CB *m*_*j*_ = +1/2 (−1/2) state via right-handed (left-handed) circularly polarized light σ^+^ (σ^–^), carrying an angular moment of +ℏ, Δ*m*_*j*_ = +1 (−ℏ, Δ*m*_*j*_ = −1),^32^ will lead to J-polarized electrons which are not spin pure, due to strong SOC, but contain a ratio of 2:1 or 1:2 spin up/down.^2^

**Figure 2:**
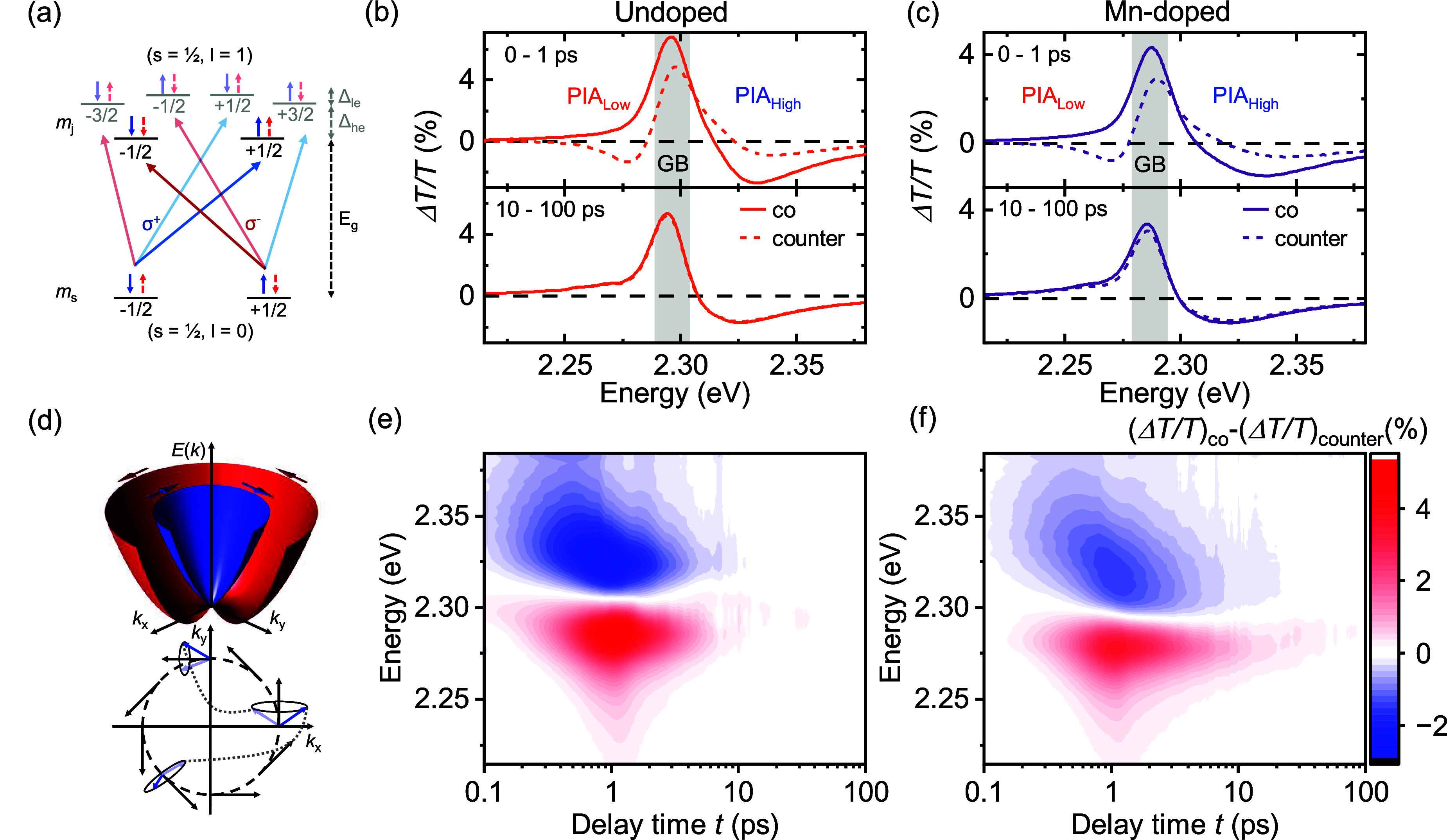
Differential circularly polarized transient absorption spectra, optical selection rules, and spin relaxation mechanism. (a) Possible angular momentum-selective optical transitions for a perovskite with an s-like valence band edge and p-like conduction band edge in the presence of reduced crystal symmetry. (b,c) Averaged CTA spectra for the two time intervals 0–1 ps (upper panel) and 10–100 ps (lower panel) for undoped and Mn-doped MAPbBr_3_, respectively. Negative feature on the low energy side PIA_Low_ and on the high energy side PIA_High_ for counter- and copolarized configurations indicates a red shift of electrons with antiparallel total angular momentum J-alignment and a blue-shift of electrons with parallel J. Difference of co- and counter-polarized CTA spectra persists longer in Mn-doped MAPbBr_3_ which indicates the influence of Mn-dopants on the spin relaxation mechanism in the material. (d) Schematic drawing of the Rashba effect creating an effective magnetic field acting on the spins which relax via motional narrowing, i.e., precessional motion between momentum scattering. (e,f) Polarization map (subtraction of CTA map under copolarized combination of pump and probe and CTA map with counter-polarized combination) of undoped MAPbBr_3_ (e) and nominally 50% Mn-doped MAPbBr_3_ (f) polycrystalline thin films at 5 K with an excitation energy of ∼2.41 eV (515 nm) and moderate fluence of 2.24 μJ/cm^2^.

CTA spectra for undoped and Mn-doped MAPbBr_3_ are shown (Figure 2b,c) for a pump fluence of 2.24 μJ/cm^2^ for copolarized combination of pump and probe (solid line) and counter-polarized combination (dashed line) for time intervals of 0–1 ps (upper panel) and 10–100 ps (lower panel) at 5 K. The spectra for Mn-doped MAPbBr_3_ are red-shifted by 10 meV with respect to the undoped material, but both spectra exhibit the same three main features: The ground state bleach (GB) marked by the gray shaded area, the negative feature on the low energy side (PIA_Low_) which is only present in the counter-polarized configuration and another negative feature (PIA_High_) on the high energy side which is much more pronounced for the copolarized combination of pump and probe.^30^ The origin of the two photoinduced absorption (PIA) features is an optically induced transient dichroism red- (blue-) shifting the absorption edge in the presence of a circularly polarized excitation with counter- (co-) polarized probe since an antiparallel alignment of spin-polarized electrons is energetically favored compared to parallel alignment (see Supporting Figure S11 for simulations).^30,33^ This difference in spectra in time corresponds to the lifetime of the net spin polarization of the charge carriers. Figure 2e,f shows the time- and spectrally resolved polarization maps ((Δ*T*/*T*)_co_ – (Δ*T*/*T*)_counter_) for both materials at a fluence of 2.24 μJ/cm^2^, which already indicate a longer living polarization of the charge carriers in the case of the Mn-doped material.

For a detailed analysis of the polarization dynamics, we evaluated the kinetics for the CTA spectra taken with co- and counter-polarized combinations of pump and probe at the GB and the PIA_Low_ feature (Figure 3a,b) for a fluence of 0.25 μJ/cm^2^. Subtraction of co- and counter-polarized transient absorption (TA) kinetics (Figure 3c) results in polarization kinetics which can be well fitted with a biexponential decay function giving two time constants. We assign the initial very fast decay, which is the same for the undoped and Mn-doped materials, to the fast spin relaxation of holes.^2^ It ranges between 0.5 and 3.5 ps for fluences from high to low, respectively (see Supporting Figure S6 for details of the holes contributing to the polarization dynamics). The second relaxation we refer to as the spin relaxation time τ_s_ which is clearly longer in the case of the Mn-doped material.

**Figure 3:**
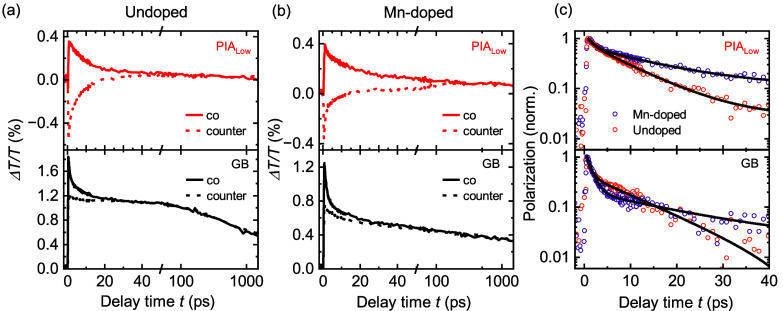
Kinetics of the GB and the PIA_Low_ features of the CTA spectra for a very low fluence of ∼0.25 μJ/cm^2^ at 5 K for undoped (a) and Mn-doped MAPbBr_3_ (b). Subtraction of co- and counter-polarized combinations of pump and probe for these two features leads to a measure for the polarization dynamics in both materials (c). The polarization can be well fitted with a biexponential decay function.

The research on revealing the different and dominating spin relaxation mechanisms in perovskites is still in progress because of its high complexity and dependency on many parameters like the type of perovskite, temperature, excitation density, and excess energy. Based on extensive literature research^16,20,32,34−37^ and our data on the temperature dependent spin dynamics in MAPbBr_3_ (Figure 4a), we identify that in this material the D-P mechanism is the dominant spin relaxation mechanism at cryogenic temperatures and low excitation densities. The origin of this effect is the presence of a k-dependent effective magnetic field due to inversion symmetry breaking in materials with strong spin–orbit coupling (SOC), first described in III–V semiconductors, also known as the Rashba^38^ and Dresselhausen^39^ effect. It was already shown that the D-P mechanism is the dominating spin relaxation mechanism for a small fluence regime in 2D perovskites like (BA)_2_MAPb_2_I_7_^35^ but also in some 3D perovskites like MAPbBr_3_^34^ which is being discussed here. While there is experimental evidence for the existence of a very strong Rashba splitting in MAPbBr_3_^37^ its origin is still under heavy debate. At cryogenic temperatures, MAPbBr_3_ presents an orthorhombic structure of space-group *Pnma*, which is centrosymmetric, with a direct bandgap located at the center of the Brillouin zone.^40^ There are several possible explanations for the observed inversion symmetry breaking, for example, defects,^41^ inversion symmetry breaking at grain boundaries, at the surface, or due to the rotation degree of freedom of the MA^+^ cation.^42,43^ While the spin relaxation time is directly proportional to the scattering time for the E-Y mechanism,^18^ it relates inversely proportional for the D-P mechanism and the spin relaxation rate can be described by
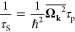
1with τ_p_ being the carrier scattering time, **Ω**(**k**) the effective magnetic field, and *ℏ* the Planck constant. This equation describes spin relaxation taking place between the scattering events during the precession around **Ω**(**k**). Since the effective magnetic field **Ω**(**k**) is **k**-dependent, its magnitude and direction change as carriers scatter from one **k** value to another (Figure 2d). The photoexcited carriers’ spins therefore experience different effective magnetic fields during their lifetime. According to the central limit theorem,^44^ the time-averaged effective magnetic field has less variation which leads to an extended spin lifetime due to suppression of dephasing of the spin ensemble. This effect was first observed in nuclear magnetic resonance (NMR) measurements of liquids and was termed motional narrowing.^45^

The D-P and E-Y mechanisms both show a nontrivial temperature-dependence which can be used to identify the dominating spin relaxation mechanism. While in case of E-Y the spin lifetime should be proportional to , it scales with  for the D-P mechanism, due to the temperature dependence of **Ω**(**k**) (for details, please see the Supporting Information).^46^ In lead halide perovskites, it was shown that the momentum scattering time τ_p_(*T*) scales with *T*^*m*^ with *m* in the range of −1.4 to −2.5.^47 ,48^ Hence, *τ*_S_ will scale with temperature between  and  and with  to  in the case of the E-Y and the D-P mechanism, respectively. Figure 4a shows the relative spin lifetime of MAPbBr_3_ as a function of temperature with a power law fit for the orthorhombic crystal structure (blue shaded) revealing a temperature dependence of *τ*_*S*_ ∝ *T*^–0.55^, which clearly points toward the D-P mechanism.

We then analyzed the fluence dependence of the polarization kinetics for the PIA_Low_ feature between ∼0.25 and 20 μJ/cm^2^ (Figure 4c). Due to polarization-dependent spectral shifts of the absorption edge which give rise to the PIA_Low/High_ features, the GB cannot be used to reliably extract the polarization dynamics. The dynamics of the polarization evaluated at the GB are not purely given by the phase space filling but are superimposed by the time-dependent spectral shift of the GB.

These superimposed spectral dynamics of the GB were extracted by a fitting routine (Supporting Figure S7) and are exemplarily shown in Figure 4b for a fluence of ∼0.25 μJ/cm^2^ for both materials. It clearly highlights the long-lived spin polarization of the Mn-doped material. We suggest that tracking of the spectral position of the GB for co- and counter-polarized states with subsequent subtraction to extract the polarization dynamics is a valid and noise-reduced method to obtain the spin relaxation times with CTA. The spin relaxation times extracted by this method (see Supporting Figure S7, spin relaxation times are plotted as a function of fluence) match the trend depicted in Figure 4c. For the high fluences (6.7 and 20 μJ/cm^2^) the spin relaxation times are the same within the errors for pristine and Mn-doped MAPbBr_3_ of around τ_S_ = 6 ps because the spin dynamics are dominated by multibody interactions.^30^ Reduction of the pump fluence leads to a change of the spin relaxation regime, and the D-P mechanism becomes dominant. Differences in the spin relaxation time between pristine and Mn-doped MAPbBr_3_ start to manifest. While τ_S_ doubles for pristine MAPbBr_3_ from around 6 ps (high fluence) to 12 ps (low fluence) it increases by a factor of 5 for the Mn-doped MAPbBr_3_ to 30 ps. Further reduction of the fluence (down to 0.25 μJ/cm^2^) levels out the spin lifetime in the case of the undoped material and even reduces the spin lifetime in the case of the Mn-doped material. According to the D-P mechanism the spin lifetime should further decrease with decreasing carrier densities due to reduced carrier–carrier scattering.^35^

Following eq 1, the precessional spin relaxation via the D-P mechanism depends on the variation of two parameters: the effective magnetic field strength **Ω**(**k**) and the momentum scattering time τ_p_. Thus, we now aim to determine the dominant factor leading to our observed extended spin lifetimes. The cooling process of the electrons due to scattering events and therefore a measure of the momentum scattering time τ_p_ can be obtained by the analysis of the initial broadening of the high energy shoulder of the GB in linearly polarized TA spectra (Figure 5a) of pristine and Mn-doped MAPbBr_3_ for the first hundreds of femtoseconds up to the picosecond range.^49,50^ After an initial very fast thermalization process in the femtosecond range, photoexcited charge carriers are Boltzmann distributed and “cool” down to the band edge via different scattering mechanisms.^51^ Fitting of the high energy shoulder of TA spectra with a Boltzmann function result in charge carrier temperatures that converge toward the environmental temperature as a function of time (Figure 5b). The time evolution of the carrier temperatures can be described and fitted with biexponential decay functions resulting in two time constants. The fits were performed on the first 20 ps for both materials. For the Mn-doped sample we obtain τ_1_ ≈ 0.10 ps and τ_2_ ≈ 2.7 ps and for the pristine sample τ_1_ ≈ 0.28 ps and τ_2_ ≈ 3.0 ps. We observe overall higher scattering rates for the Mn-doped material and identify 2.8 times faster scattering for the first time constant. We attribute the two time constants to two different groups of optical phonon modes. The introduction of Mn-dopants appears to increase the density of optical phonons, particularly in the high energy mode. This difference in momentum scattering time can explain the extended spin lifetimes that we observed within the error bars. From our results, we can deduce that the spin of the Mn-dopants does not contribute to the spin relaxation mechanism of the photoexcited charges in the investigated fluence regime. Considering the quantitative agreement of changes in spin lifetimes and momentum scattering times, the dominating process is the enhanced motional narrowing induced by the dopants. Further small contributions to the extended spin lifetimes in Mn-doped samples could arise from changes in the Rashba parameter to some extent. The exact mechanism of the dopants influencing the momentum scattering time with respect to different dopants, their concentration and size, and other parameters like the morphology, grain size, and boundaries will be investigated in the future work. We have first evidence that our mechanism is applicable to other (nonmagnetic) dopants (Supporting Information Figure S10). A mechanism which was reported in a variety of dilute magnetic semiconductors^52,53^ and also in a first study of Mn-doped perovskite microrods^54^ is the formation of exciton-magnetic-polarons (EMP) which could potentially stabilize the charge carriers’ spin. Signatures of the formation of EMPs in the Mn-doped material can be found in micro-PL measurements for high excitation densities and cryogenic temperatures (Supporting Figure S9). Due to the absence of this feature in the CTA spectra and the orders of magnitude slower EMP formation time^55^ compared to the spin lifetime, we can exclude this mechanism as origin for our observed extended spin lifetimes. Yet, if EMP formation times and spin lifetimes reached a common regime, we would expect dramatically extended spin relaxation times.

**Figure 4:**
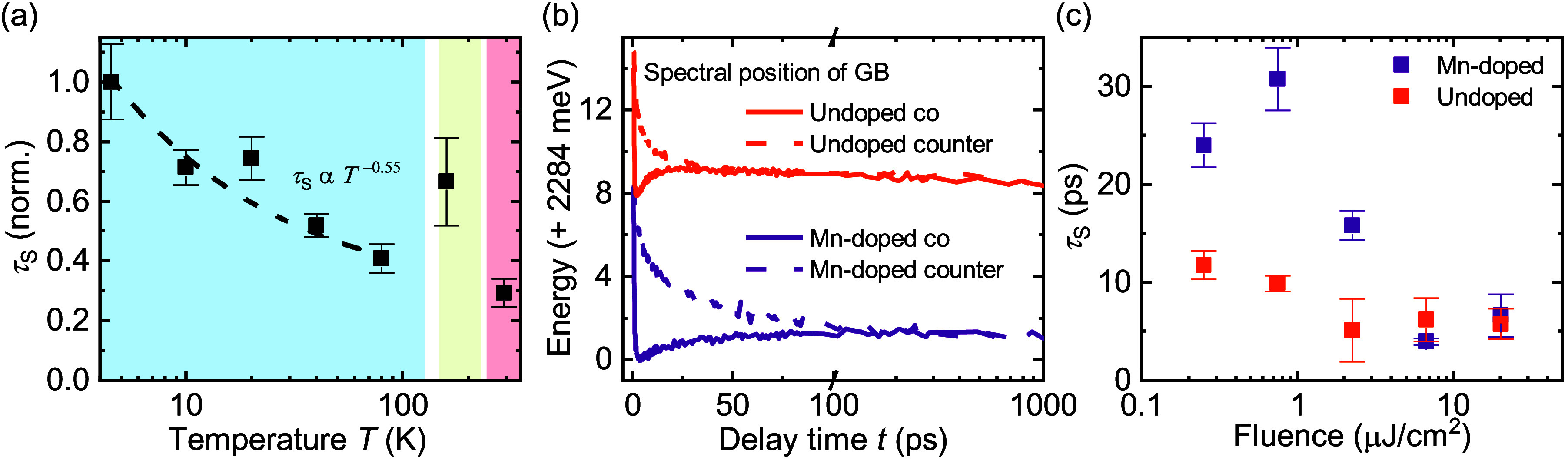
(a) Relative spin lifetime of MAPbBr_3_ for a pump fluence of ∼0.2 μJ/cm^2^ as a function of temperature. The shaded areas indicate the three different crystal structures MAPbBr_3_ forms depending on the temperature (from left to right: blue - orthorhombic, green - tetragonal, red - cubic). The fit with a power law of the spin lifetime as a function of temperature is shown with the dashed line for the orthorhombic phase. (b) Optically induced circular dichroism, which is responsible for the emergence of the PIA_Low_/PIA_High_ features, also leads to a shift of the GB in energy. The kinetics of this splitting, measured for a very low fluence of ∼0.25 μJ/cm^2^, display the polarization dynamics extracted at the PIA_Low_ feature. The spin relaxation times plotted in (c) were extracted by a biexponential fit from the polarization dynamics obtained at the PIA_Low_ feature as a function of the excitation fluence for undoped and Mn-doped MAPbBr_3_. Since the ultrafast initial decay is the same for both materials and all fluences, only the second time constant referred to as the spin relaxation time τ_S_ is plotted.

**Figure 5:**
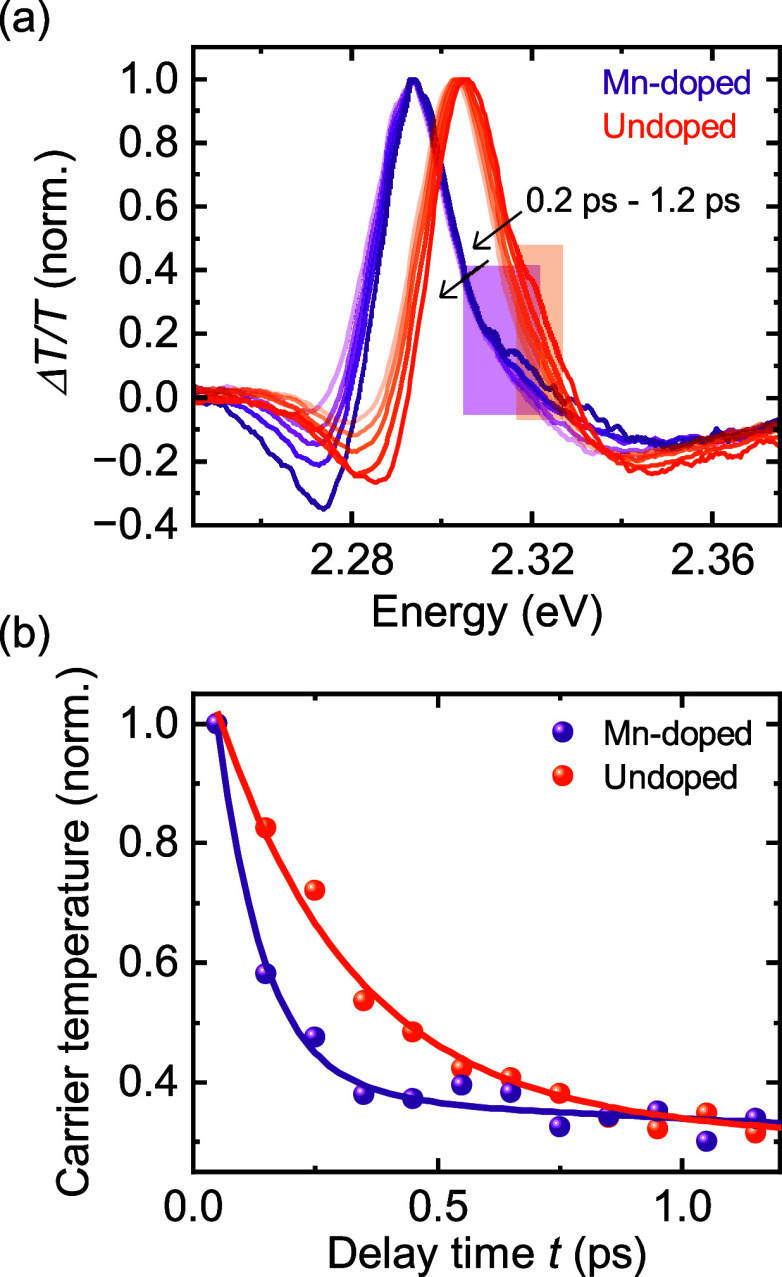
(a) Normalized linearly polarized TA spectra of undoped and Mn-doped MAPbBr_3_ for the first hundreds of femtoseconds up to 1.2 ps for a fluence of 0.75 μJ/cm2 at 5 K. The colored boxes depict the energy regimes for the fit of the high energy shoulder with a Boltzmann distribution. (b) Extracted normalized carrier temperatures as a function of delay time fitted with a biexponential function.

In conclusion, we presented the first investigation of the influence of high-level paramagnetic doping on the spin lifetime in bulk hybrid metal halide perovskites, exploiting the technique of broadband CTA. We have shown that Manganese doping of solution-processed perovskite thin films of MAPbBr_3_ extended the spin lifetime of photoexcited charge carriers. The quantitative study of the momentum scattering rates revealed faster scattering in the presence of the Manganese dopants. Therefore, we attribute the extended spin lifetimes to enhanced motional narrowing effects because of higher momentum scattering rates, which influence the dominating spin relaxation process, the D-P mechanism.

## Data Availability

Data files presented in this manuscript can be found at https://doi.org/10.11588/data/VM8ZKT.

## Supporting Information Available

Experimental details, characterization methods, details on simulations, data processing and calculations; additional data on temperature dependent micro-PL and on the spin dynamics of Mg-doped MAPbBr_3_ (PDF)
