# Supplementary material for: Motional Narrowing Effects in the Excited State Spin Populations of Mn-Doped Hybrid Perovskites
Source: J Phys Chem Lett. 2024 Mar 5;15(10):2851–8. doi: 10.1021/acs.jpclett.3c03466 (PMC10945573; doi:10.1021/acs.jpclett.3c03466)
Supplement: Supplementary file 1 — jz3c03466_si_001.pdf [file jz3c03466_si_001.pdf]

Supporting Information

Motional Narrowing Effects in the Excited State Spin Populations of Mn-Doped Hybrid Perovskites

Jonathan Zerhoch,[1,2,3] Stanislav Bodnar,[1,2,3] James E. Lerpinière,[4] Shangpu Liu,[1,2,3] Timo Neumann,[2,3,5] Barbara Sergl,[2,3] Markus W. Heindl,[1,2,3] Andrii Shcherbakov,[1,2,3] Ahmed Elghandour,[6] Rüdiger Klingeler,[6] Alison B. Walker,[4] Felix Deschler[1*]

[1]Physikalisch-Chemisches Institut, Universität Heidelberg, Im Neuenheimer Feld 229, 69120 Heidelberg, Germany

[2]Walter Schottky Institut, Technische Universität München, Am Coulombwall 4, 85748 Garching, Germany

[3]Physics Department, TUM School of Natural Sciences, Technische Universität München, Am Coulombwall 4, 85748 Garching, Germany

[4]Department of Physics, University of Bath, Bath BA2 7AY, UK

[5]Cavendish Laboratory, University of Cambridge, JJ Thomson Ave, Cambridge CB3 0HE, UK

[6]Kirchhoff Institut für Physik, Universität Heidelberg, Im Neuenheimer Feld 227, 69120 Heidelberg, Germany

Experimental Methods

Synthesis of polycrystalline thin films of $MAPbBr_3$ and Mn-doped $MAPbBr_3$

All chemicals were purchased from Sigma-Aldrich and were used without further purification. Standard solution processing procedure of the precursors was used and all steps were performed in a nitrogen-filled glovebox. MABr, Pb(II)-acetate trihydrate and Mn(II)-acetate tetrahydrate were dissolved in DMF with a 0.2 M concentration at room temperature for 1 h under continuous stirring. Following the recipe described in the work of Lukas M. Falk et al.[1], the precursor solutions were mixed in a 3:1 ratio of MABr to Pb-acetate with Mn-acetate replacing Pb-acetate to achieve the desired doping level. Then 100 µl of the perovskite precursor solution was spin-coated with 5000 rpm for 30 s on an O_2-plasma-treated glass substrate with subsequent annealing at 80°C for 15 min.

EDX

For investigations of the atomic composition of the doped sample a Bruker XFlash6130 energy-dispersive X-ray spectrometer, mounted in a Zeiss EVO MA10 for sample alignment imaging, was used. For this measurement the sample was spin-coated on an FTO substrate for better conductivity to prevent charging of the sample.

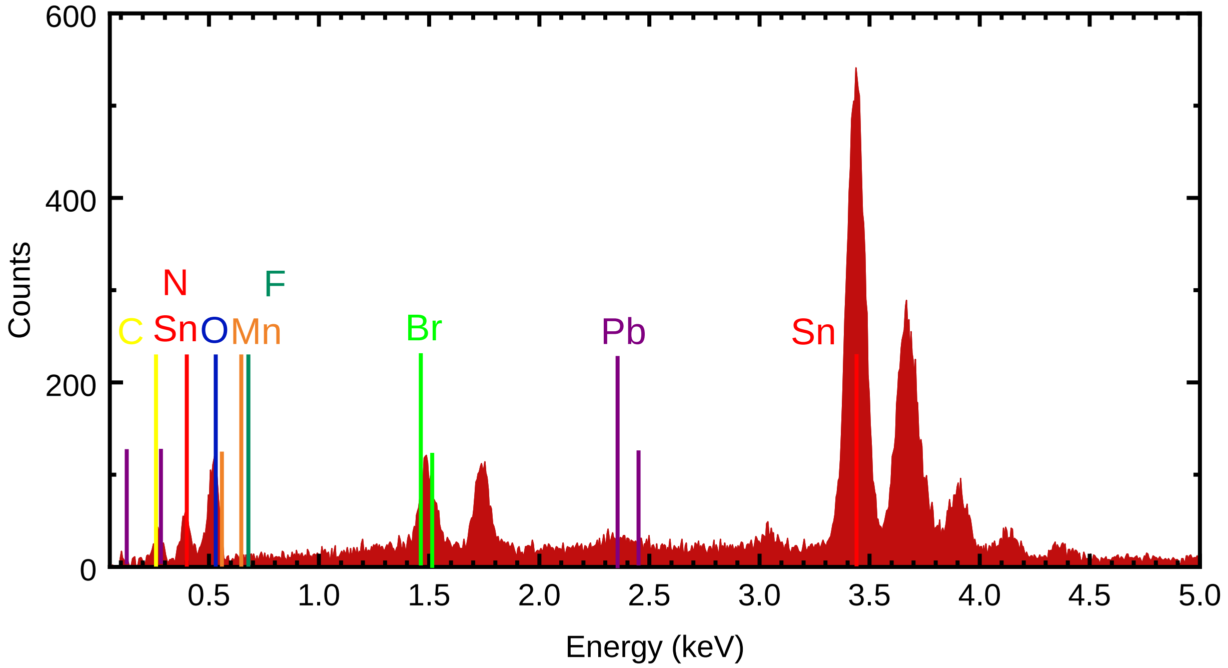

Supporting Figure S1: EDX spectrum of Mn-doped $MAPbBr_3$ (50 % nominal doping level).

Supporting Table T1: Atomic ratios and mass ratios obtained by the series fit deconvolution.

Element	At. No.	Netto	Mass [%]	Atom [%]	Abs. error [%] (1 sigma)	Rel. error [%] (1 sigma)
Carbon	6	80	1.64	5.71	0.11	6.52
Nitrogen	7	194	4.15	12.42	0.22	5.38
Oxygen	8	1049	20.09	52.62	1.02	5.05
Fluorine	9	51	1.44	3.17	0.10	6.91
Manganese	25	78	0.82	0.62	0.05	5.68
Bromine	35	54	5.50	2.88	0.16	2.96
Tin	50	19079	60.64	21.41	1.63	2.69
Lead	82	71	5.74	1.16	0.17	2.94
		**Sum**	**100.00**	**100.00**		

Magnetization measurements

The magnetization measurements were performed at a superconducting quantum interference device (SQUID, MPMS3 Quantum design) capable of temperatures down to 1.8 K and magnetic fields up/down to ± 7 T.

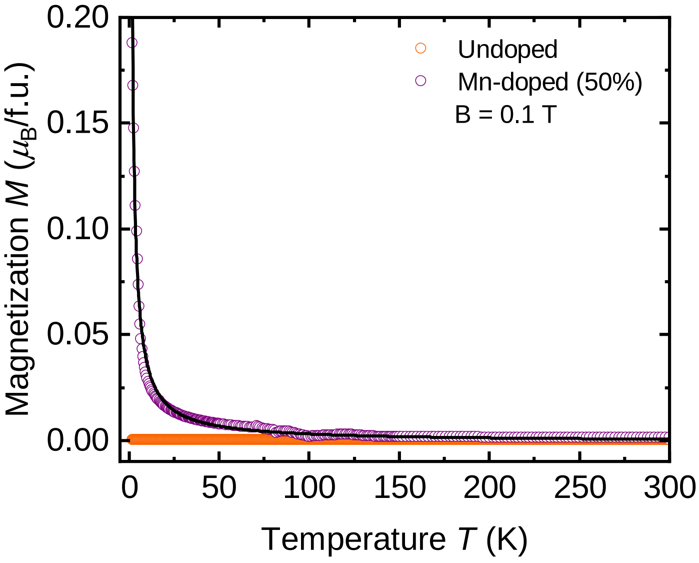

Supporting Figure S2: Magnetization measurements of pristine and Mn-doped $MAPbB_{r3}$ in presence of an external magnetic field of 0.1 T. The nominal doping level 50% corresponds to the doping level in the precursor solution. Undoped $MAPbBr_3$ shows a very weak paramagnetic response ($\chi \approx 1*10^{-3}$ erg/G^2mol). The Mn-doped material shows dominant Curie paramagnetism for low temperatures, which is indicated by the fit of the data with the Curie law (black line). A shoulder around 90 K and 125 K for the 50% Mn-doped sample could indicate the existence of an additioal Mn-phase like MnO or MnO_2. No additional magnetic transitions which would imply the existence of other magnetic impurities and clusters can be observed.

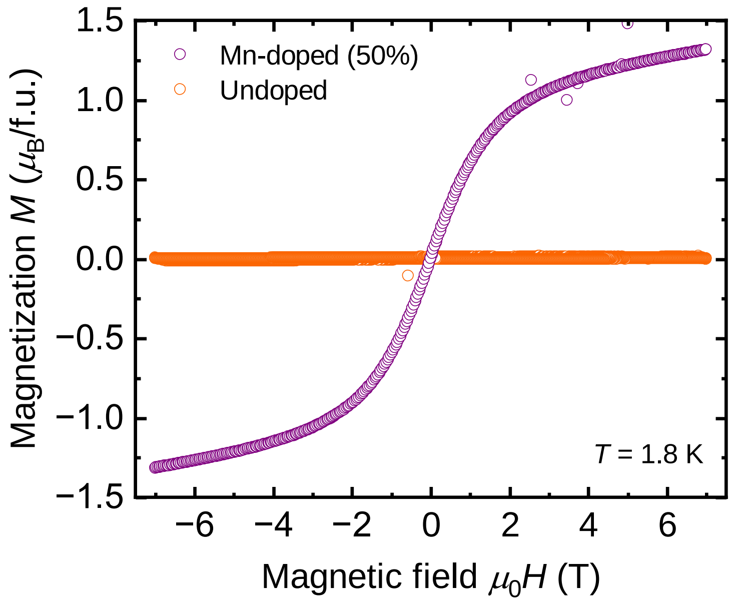

Supporting Figure S3: Magnetization measurements as a function of the external magnetic field of pristine and Mn-doped $MAPbBr_3$ at 1.8 K. Magnetization of the undoped material shows a very weak positive magnetic susceptibility while the Mn-doped material shows a pronounced paramagnetic response.

Quantification of Mn content:

The EDX measurements showed that the actual Mn content in the thin film, starting from a precursor solution in which 50% of the lead was replaced by Manganese, was about 35%. Since EDX measurements are not sensitive to the valence state of the elements, magnetization measurements were performed to find a lower limit for the Mn^{2+} content in the nominally 50 % Mn-doped material. The magnetization shown in Figure 1e in the main text was fitted with the function

$$M = M_S B_{\frac{5}{2}}(x) + \chi_0 \mu_0 H \tag{1}$$

with the saturation magnetization

$$M_S = N_{dopants} g \mu_B J \tag{2}$$

where $N_{dopants}$ stands for the number of free moments with total magnetic moment J per formula unit which here implies the number of Mn^{2+} ions, the Brillouin function describing a system with a total angular momentum J

$$B_J(x) = \frac{2J+1}{2J} \coth\left(\frac{2J+1}{2J} x\right) - \frac{1}{2J} \coth\left(\frac{1}{2J} x\right) \tag{3}$$

and x being

$$x = J \frac{g \mu_B \mu_0 H}{k_B T} \tag{4}$$

where the g-factor was assumed to be 2,[2] the Bohr magneton μ_B, the magnetic field strength $\mu_0 H$, the Boltzmann constant k_B, and the temperature T. An additional linear term $\chi_0 \mu_0 H$ was required to account for a linear contribution to the total magnetization.

The presence of a linear term may be interpreted as signature for an antiferromagnetic contribution. The fit results in $N_{dopants} = 0.183 \frac{1}{f.u.}$ (18.34 %) for the nominally 50 % Mn-doped material. We conclude that at least a minimal doping concentration of 18.34 % of Mn^{2+} ions are present. The linear contribution is in the range of $\chi_0 = 0.06 \pm 0.001 \frac{\mu_B}{f.u.*T}$. Slight deviations of the data from the $B_{\frac{5}{2}}(x)$ Brillouin function could indicate the presence of paramagnetic Mn^{3+}/Mn^{4+} ions in an impurity phase, to some extent.

Ultrafast broadband circularly polarized transient absorption spectroscopy (CTA)

All transient absorption measurements were conducted at a home-built pump-probe setup with the sample being located inside a closed-cycle magneto-optic cryostation (Montana Instruments). The CTA measurements were performed at 5 kHz repetition rate of the laser system (Pharos by Light Conversion). The fundamental of 1030 nm was frequency-doubled and also used to generate white light (Hiro by Light Conversion). The 515 nm beam was running via a delay stage (Newport DLS325) used to pump the material and chopped down to 2.5 kHz while the generated white light was used to probe in the spectral region of interest detected by a spectrometer with a mounted sCMOS camera (Kymera 193i and Zyla 5.5 by Andor Oxford Instruments). Pump and probe were both circularly polarized by the same superachromatic quarter-wave plate (specified for 310 – 1100 nm by B-Halle) right in front of the entrance window of the cryostation. Switching between co- and counter polarized combinations of pump and probe was achieved via rotation of the linearly polarized pump with a superachromatic half-wave plate (specified for 310 – 1100 nm by B-Halle) before passing the quarter wave plate.

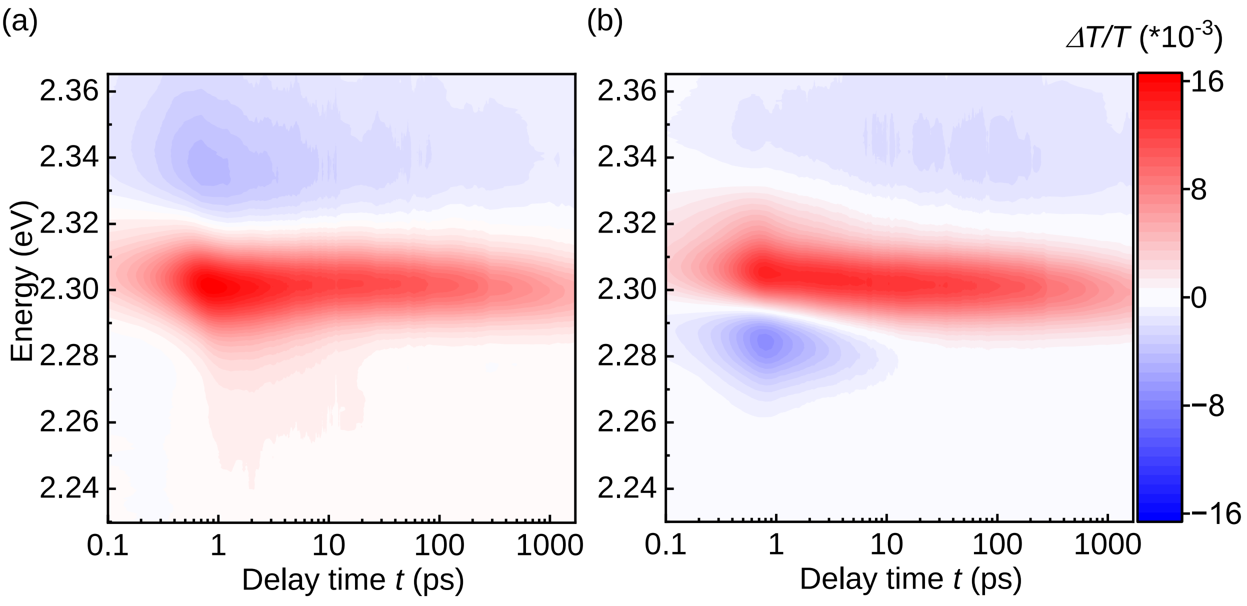

Supporting Figure S4: Differential circularly polarized transient absorption map of $MAPbBr_3$ at 5 K with a fluence of ~0.75 µJ/cm^2 and excitation energy of ~2.41 eV for co- and counter-polarized combinations of pump and probe plotted in

(a) and (b), respectively. Co-polarized map shows distinct PIA feature on the high energy side while counter-polarized map shows PIA feature on the low energy side.

Spin relaxation mechanism:

Spin relaxation mechanisms of excited charge carriers in semiconductors can be very complex and depend on numerous parameters like the material itself (crystal symmetry, spin-orbit coupling, quantum confinement…), temperature, charge carrier density and excess energy. Depending on these parameters one mechanism can be more dominant than the other and via changing one or more of these parameters the material can be tuned from one spin relaxation regime to another. Most research on this topic was done for III-V inorganic semiconductors. Except for the charge carrier recombination which is also one way of spin relaxation that usually happens orders of magnitude slower than the actual spin relaxation, three main relaxation mechanisms were identified. The Elliot-Yafet (E-Y), the D'yakonov Perel (D-P) and Bir-Aronov-Pikus (BAP) effect. The BAP mechanism describes spin relaxation in p-doped semiconductors or in confined systems, where spin flip happens via Coulomb exchange coupling between electrons and holes. This effect can be neglected for $MAPbBr_3$. Concerning the E-Y mechanism scattering events of the charge carriers with phonons or impurities e.g. cause spin flip. For III-V semiconductors and the E-Y mechanism, the spin lifetime can be written as[3]

$$\frac{1}{\tau_S^{EY}} = A\left(\frac{k_B T}{E_g}\right)^2 \eta^2 \left(\frac{1-\frac{\eta}{2}}{1-\frac{\eta}{3}}\right)^2 \frac{1}{\tau_p} \quad (5)$$

where τ_p is the momentum relaxation time, E_g the band gap, $\eta = \Delta/(E_g + \Delta)$ with the spin-orbit splitting Δ, and A a dimensionless constant. In the D-P mechanism spins relax due to precessional motion in the presence of effective magnetic fields caused by the Dresselhaus or Rashba effect. Momentum scattering between the precession prevents spins from relaxation. The spin lifetime can be written as[3]

$$\frac{1}{\tau_S^{DP}} = Q\alpha^2 \frac{(k_B T)^3}{\hbar^2 E_g} \tau_p \quad (6)$$

with Q a dimensionless constant and α given by

$$\alpha \simeq \frac{4\eta}{\sqrt{3-\eta}} \frac{m_c}{m_0} \quad (7)$$

where m_c and m_0 are the effective mass of the electron and the free electron mass, respectively. We see that both E-Y and D-P effect show a strong temperature dependence which is not trivial because of the momentum scattering time that also depends strongly on the temperature. To summarize, the behaviors for both mechanisms depend on the temperature and the spin lifetimes are directly and inversely proportional to the momentum scattering time τ_p for the E-Y and the D-P mechanism, respectively. In the E-Y mechanism, with decreasing temperature the spin lifetime should increase proportional to $\frac{\tau_p(T)}{T^2}$, while for the D-P mechanism the spin lifetime should increase proportional to $\frac{1}{T^3 \tau_p(T)}$. In case of the D-P mechanism the increase of the spin lifetime with decreasing temperature will be slowed down by the increase in the momentum scattering time so that the overall temperature

dependence deviates strongly from the T^3 behavior. In lead halide perovskites it was shown that the momentum scattering time $\tau_p(T)$ scales with T^m with m in the range of -1.4 to -2.5.[4,5] Considering the two extreme cases with m = -1.4 and m = -2.5, τ_S^{EY} should be proportional to $\frac{1}{T^{3.4}}$ and $\frac{1}{T^{4.5}}$,, while τ_S^{DP}should be proportional to $\frac{1}{T^{1.6}}$ and $\frac{1}{T^{0.5}}$, respectively. Figure S5 shows the relative spin lifetime change as a function of temperature. The spin lifetime increases by a factor of three from room temperature to 4.5 K. Connecting the relative change in spin lifetime to the crystal structure one can clearly see the crystal phase change from cubic to tetragonal to orthorhombic which leads to a decrease of the spin lifetime (around 150 K) that can be traced back to a further reduction in symmetry of the crystal structure, probably transition from on spin relaxation regime to another (E-Y to D-P). A power law fit (dashed line in Figure S5) of the spin lifetime for the orthorhombic phase results in $\tau_S(T)\ \alpha\ T^{-0.55}$, clearly underlining D-P as the dominating spin relaxation mechanism in this regime.

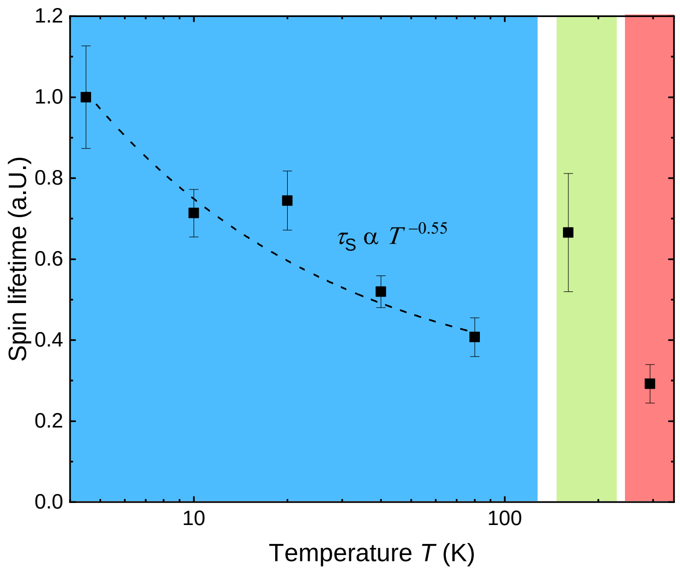

Supporting Figure S5: Spin lifetime of $MAPbBr_3$ normalized to the maximum spin lifetime at 4.5 K with a pump fluence of ~0.2 μJ/cm^2 as a function of temperature. The shaded areas indicate the three different crystal structures $MAPbBr_3$ forms depending on the temperature[6] (from left to right: blue - orthorhombic, green - tetragonal, red - cubic). The fit with a power law of the spin lifetime as a function of temperature is shown with the dashed line for the orthorhombic phase.

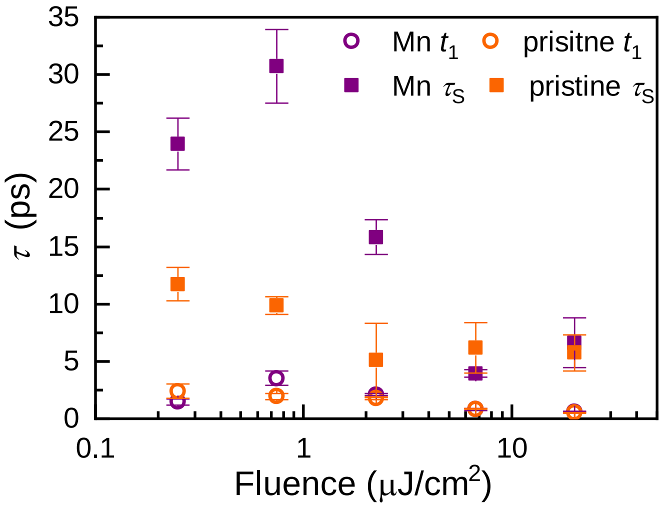

Supporting Figure S6: Time constants obtained from polarization dynamics by biexponential fits. The first initial fast decay t_1 is assigned to the hole spin lifetime, the second time constant τ_s to the spin lifetime of electrons. The hole spin lifetimes of the pristine and Mn-doped materials increase similarly with decreasing fluences. Due to the s-like valence band, holes are most likely less affected by the SOI leading to a reduced impact of the Mn-doping to the holes' spin via the D-P mechanism.

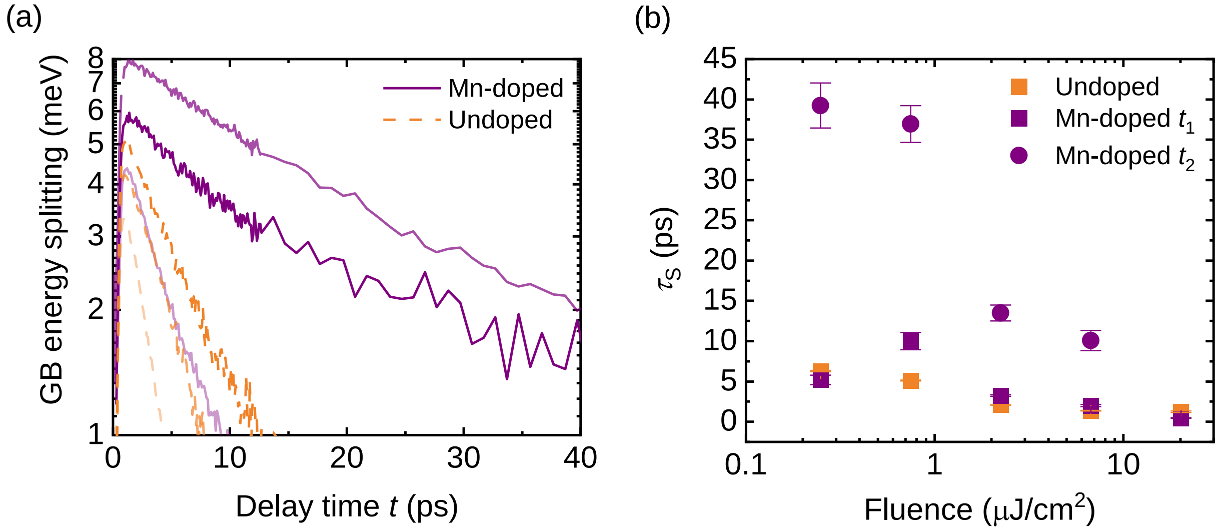

Supporting Figure S7: (a) The optically induced circular dichroism, which is responsible for the emergence of the PIA_{Low}/PIA_{High} features also leads to a shift of the GB in energy. The splitting in energy of the GB between co- and counter-polarized combinations of pump and probe is plotted as a function of delay time for Mn-doped and undoped $MAPbBr_3$ for three different fluences. The fluences ~(0.25; 0.75; 2.25) μJ/cm^2 are represented by the intensity of the color-plot from dark to light, respectively. The position of the GB was extracted with a fitting routine which locally fits the GB peak and finds the position of the maximum. The kinetics of the GB-splitting were fitted with mono-exponential (undoped $MAPbBr_3$) and bi-exponential (Mn-doped $MAPbBr_3$) functions. The obtained time constants are depicted in (b). The Mn-doped material shows a second long-lived spin lifetime component t_2 compared to the undoped material.

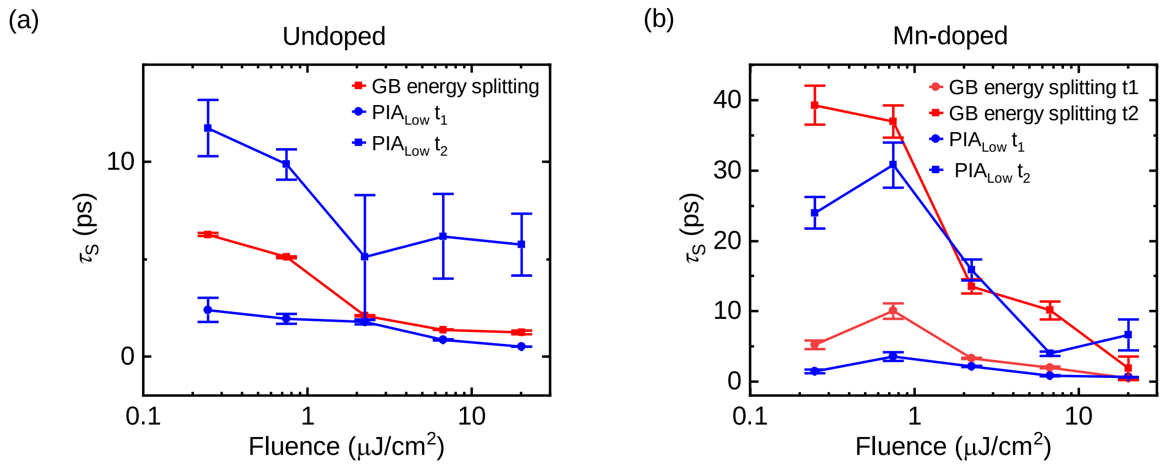

Supporting Figure S8: Direct comparison of the spin lifetimes extracted by the PIA_{Low} feature and the transient energy splitting of the GB between co and counter polarized spectra for undoped (a) and Mn-doped $MAPbBr_3$ (b). In case of the undoped material the GB energy splitting can be perfectly fitted with a mono-exponential function while the polarization extracted by the PIA_{Low} feature scales more bi-exponential. As expected, the spin lifetimes for the GB energy splitting method are between t_1 and t_2, but follow the same trend pretty well. In case of the Mn-doped sample the comparability is better since the polarization dynamics extracted from both methods follow a bi-exponential function, resulting in the same overall trend with overall slightly higher lifetimes in case of the GB energy splitting method.

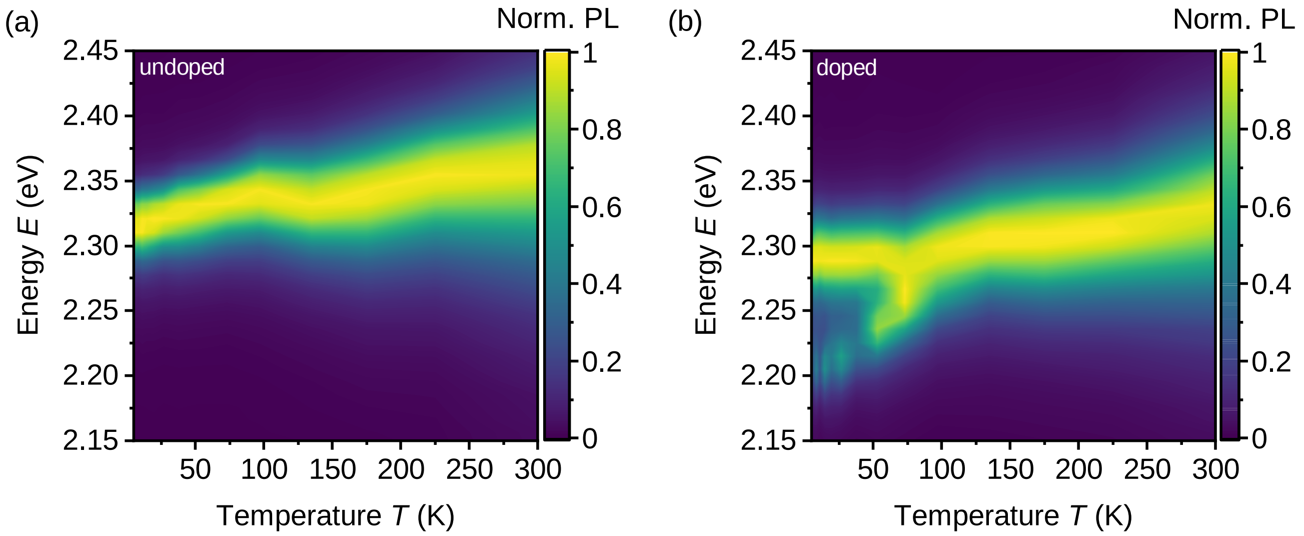

Supporting Figure S9: Micro-PL measurements as a function of temperature at a very high fluence (~400 μJ/cm^2) excited at 2.76 eV for undoped (a) and nominally 50 % Mn-doped $MAPbBr_3$ (b).

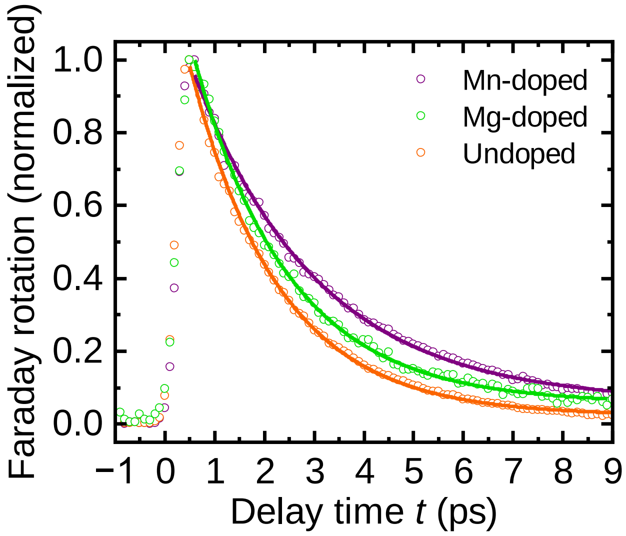

Supporting Figure S10: Faraday rotation measurements at 5 K pumped at ~2.41 eV (515 nm) and probed at ~1.77 eV (700 nm) for nominally 50 % Mn-doped, Mg-doped and undoped $MAPbBr_3$ thin films in a moderate fluence regime. The Mg-doped sample was prepared with the same recipe, mentioned in the synthesis sections, and the use of a Mg-acetate tetrahydrate precursor.

CTA simulations

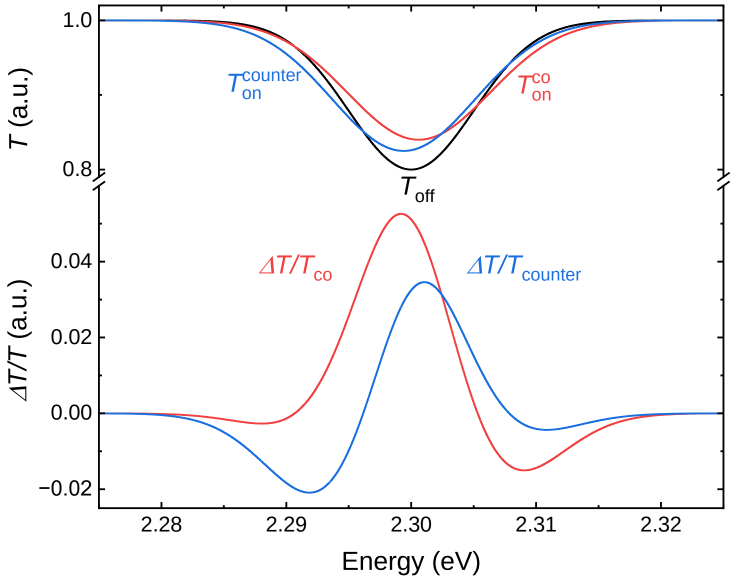

Supporting Figure S11: Simulation of the CTA spectra for co- and counter-polarized combinations of pump and probe which demonstrates the formation of the PIA_{High} and PIA_{Low} features. We assume an idealized transmission profile of Gaussian shape T_{off} which is modified in presence of an excitation pulse changing the amplitude of the Gaussian (due to the GB) and also its width due to changes in the dielectric function. In addition, the transmission redshifts (blueshifts) for counter- (co-) polarized combinations of pump and probe since the injection of charge carriers with antiparallel (parallel) spins is energetically preferred (avoided) which leads to a redshift (blueshift) of the transmission profile. The simulated TA spectra obtained via $\frac{T_{on}-T_{off}}{T_{off}}$ are shown for co- and counter-polarized combinations of pump and probe.

Quantitative analysis of charge carrier cooling

We make the usual assumption, when analyzing TA experiments, that the differential transmission at energy ε, $\Delta T/T(\varepsilon)$, is proportional to the carrier population at this energy, $f(\varepsilon)$, which essentially amounts to neglecting the contribution to the signal from the photoinduced refractive index change of the sample. Since we restrict our fit to the high-energy tail of the GB, we approximate $f(\varepsilon)$ by a Maxwell-Boltzmann distribution. Hence, we fit to our signal a function of the form:

$$\frac{\Delta T}{T}(\varepsilon) \propto A_1\sqrt{\varepsilon - \varepsilon_G}\, e^{-\frac{\varepsilon-\varepsilon_F}{k_B T_c}} + A_2, \tag{8}$$

with ε_G the bandgap energy, ε_F the quasi-Fermi level, k_B Boltzmann's constant, T_c the carrier temperature and A_1 and A_2 fit constants relating to the bandfilling and bandgap renormalization contributions, respectively. We further simplify this by assuming $\varepsilon_G = \varepsilon_F$, which is commensurate with our assumption of Boltzmann statistics, so that there are four free parameters in our fits: A_1, A_2, e_F and T_c. No widespread consensus exists regarding the definition of the high-energy tail of the signal when performing this analysis; here, we define it as the signal in the energy window between, at the lower end, the energy corresponding to half the maximum bleach at a delay time of 0.35 ps and, at the higher end, by the zero-crossing of the TA signal at this delay.

It is typical to obtain a value for ε_F by fitting the function specified in (4) to the signal for the longest measured delay time, with T_c set to the lattice temperature (*i.e.*, it is assumed the carrier population has fully cooled at the longest measurement time). This, then, reduces the number of free fit parameters to three for all other delay times. However, here, we find that (4) with T_c fixed to the lattice temperature of 5 K gives a poor fit to the measured signal at the longest delay of 1686.75 ps. Instead, we set T_c to 20 K for this delay, which we find gives a much better fit to the data for both samples, obtaining values for ε_F of 2.3160 eV for the undoped sample and 2.3048 eV for the Mn-doped sample. Beyond the improved fit to our measurements, we further justify the use of the supra-lattice temperature of 20 K by making the point that, for carrier populations at such low temperatures, the number of carriers with energy above the emission threshold for even the lowest frequency optical phonon mode becomes extremely small and the carrier cooling rate becomes very slow. Thus, that the carrier population has not fully cooled even after > 1 ns is conceivable.

For all other delay times, we fix the value of ε_F in (4) to the appropriate value obtained from the fit for a 1685.75 ps delay and allow the values of A_1 and A_2 to vary ±50 % compared with the fitted values at this longest delay, which should allow for a physical variation of the amplitudes of the bandfilling and bandgap renormalization contributions with delay time. Hence, we get T_c as a function of delay time from our fits, to which we fit biexponential decays for both samples.

References:

(1) Falk, L. M.; Goetz, K. P.; Lami, V.; An, Q.; Fassl, P.; Herkel, J.; Thome, F.; Taylor, A. D.; Paulus, F.; Vaynzof, Y. Effect of Precursor Stoichiometry on the Performance and Stability of $MAPbBr_3$ Photovoltaic Devices. *Energy Technol.* **2020**, *8*, 1900737.

(2) Neumann, T.; Feldmann, S.; Moser, P.; Delhomme, A.; Zerhoch, J.; van de Goor, T.; Wang, S.; Dyksik, M.; Winkler, T.; Finley, J. J.; Plochocka, P.; Brandt, M. S.; Faugeras, C.; Stier, A. V.; Deschler, F. Manganese Doping for Enhanced Magnetic Brightening and Circular Polarization Control of Dark Excitons in Paramagnetic Layered Hybrid Metal-Halide Perovskites. *Nat. Commun.* **2021**, *12*, 3489.

(3) Song, P. H.; Kim, K. W. Spin Relaxation of Conduction Electrons in Bulk III-V Semiconductors. *Phys. Rev. B* **2002**, 66, 035207.

(4) Bourelle, S. A.; Camargo, F. V. A.; Ghosh, S.; Neumann, T.; van de Goor, T. W. J.; Shivanna, R.; Winkler, T.; Cerullo, G.; Deschler, F. Optical Control of Exciton Spin Dynamics in Layered Metal Halide Perovskites via Polaronic State Formation. *Nat. Commun.* **2022**, *13*, 3320.

(5) Wright, A. D.; Verdi, C.; Milot, R. L.; Eperon, G. E.; Pérez-Osorio, M. A.; Snaith, H. J.; Giustino, F.; Johnston, M. B.; Herz, L. M. Electron-Phonon Coupling in Hybrid Lead Halide Perovskites. *Nat. Commun.* **2016**, *7*, 11755.

(6) Liu, Y.; Lu, H.; Niu, J.; Zhang, H.; Lou, S.; Gao, C.; Zhan, Y.; Zhang, X.; Jin, Q.; Zheng, L. Temperature-Dependent Photoluminescence Spectra and Decay Dynamics of $MAPbBr_3$ and $MAPbI_3$ Thin Films. *AIP Adv.* **2018**, *8,* 095108.
